# Scaffold Geometry-Imposed Anisotropic Mechanical Loading Guides the Evolution of the Mechanical State of Engineered Cardiovascular Tissues *in vitro*

**DOI:** 10.3389/fbioe.2022.796452

**Published:** 2022-02-16

**Authors:** L. H. L. Hermans, M. A. J. Van Kelle, P. J. A. Oomen, R .G. P. Lopata, S. Loerakker, C. V. C. Bouten

**Affiliations:** ^1^ Department of Biomedical Engineering, Eindhoven University of Technology, Eindhoven, Netherlands; ^2^ Institute for Complex Molecular Systems, Eindhoven University of Technology, Eindhoven, Netherlands

**Keywords:** cardiovascular, tissue engineering, growth, remodeling, scaffold geometry

## Abstract

Cardiovascular tissue engineering is a promising approach to develop grafts that, in contrast to current replacement grafts, have the capacity to grow and remodel like native tissues. This approach largely depends on cell-driven tissue growth and remodeling, which are highly complex processes that are difficult to control inside the scaffolds used for tissue engineering. For several tissue engineering approaches, adverse tissue growth and remodeling outcomes were reported, such as aneurysm formation in vascular grafts, and leaflet retraction in heart valve grafts. It is increasingly recognized that the outcome of tissue growth and remodeling, either physiological or pathological, depends at least partly on the establishment of a homeostatic mechanical state, where one or more mechanical quantities in a tissue are maintained in equilibrium. To design long-term functioning tissue engineering strategies, understanding how scaffold parameters such as geometry affect the mechanical state of a construct, and how this state guides tissue growth and remodeling, is therefore crucial. Here, we studied how anisotropic versus isotropic mechanical loading—as imposed by initial scaffold geometry—influences tissue growth, remodeling, and the evolution of the mechanical state and geometry of tissue-engineered cardiovascular constructs *in vitro*. Using a custom-built bioreactor platform and nondestructive mechanical testing, we monitored the mechanical and geometric changes of elliptical and circular, vascular cell-seeded, polycaprolactone-bisurea scaffolds during 14 days of dynamic loading. The elliptical and circular scaffold geometries were designed using finite element analysis, to induce anisotropic and isotropic dynamic loading, respectively, with similar maximum stretch when cultured in the bioreactor platform. We found that the initial scaffold geometry-induced (an)isotropic loading of the engineered constructs differentially dictated the evolution of their mechanical state and geometry over time, as well as their final structural organization. These findings demonstrate that controlling the initial mechanical state of tissue-engineered constructs via scaffold geometry can be used to influence tissue growth and remodeling and determine tissue outcomes.

## 1 Introduction

When conventional treatments fail, end-stage diseased cardiovascular tissues like heart valves or blood vessels require replacement. If autografts are unavailable, non-living prostheses are commonly used. Such prostheses, like bioprosthetic and mechanical valves, or synthetic vascular grafts, have several shortcomings, but most importantly lack the ability to grow, repair and remodel in response to changes in their environment. This aspect is particularly problematic for pediatric patients, as it requires them to undergo multiple re-operations (Ackermann et al., 2007; Lee et al., 2011; Brown et al., 2012) that reduce life expectancy (Puvimanasinghe et al., 2001).

*In situ* tissue engineering is a promising approach to develop a new generation of cardiovascular grafts that potentially overcomes the aforementioned drawback. With this approach, a (synthetic) biodegradable scaffold is implanted that employs the regenerative capacity of the body to form a living, autologous tissue. Ideally, the resulting tissue-engineered construct should grow, repair and remodel like a native tissue (Mayer, 2001; Yacoub and Takkenberg, 2005), and therefore require no follow-up operations. Nevertheless, physiological growth and remodeling of tissue-engineered constructs remains challenging. Several studies reported that constructs became geometrically unstable after implantation as a result of adverse tissue growth and remodeling, such as aneurysm formation in vascular grafts (McAllister et al., 2009; Khosravi et al., 2016), and leaflet shortening in valvular prostheses (Schmidt et al., 2010; Driessen-Mol et al., 2014; Reimer et al., 2017). In native tissues, the outcome of growth and remodeling, either physiological or pathological, is thought to depend at least partly on the establishment of a homeostatic mechanical state (Humphrey, 2008; Cyron and Humphrey, 2014; Humphrey et al., 2014), where one or more mechanical quantities in a tissue are maintained in equilibrium. Various quantities have been suggested to define such a mechanical homeostasis in different tissues, including stress (Taber and Humphrey, 2001; Humphrey et al., 2009; Valentín et al., 2009), strain (Chaput et al., 2008; Oomen et al., 2016), strain energy density (Cyron and Humphrey, 2014; Vigliotti et al., 2016), and stiffness (Ferruzzi et al., 2016; Bellini et al., 2017). The mechanical state of tissue-engineered constructs arises from the external loading conditions, their geometry and mechanical properties. We therefore believe that by understanding the interplay between tissue growth, remodeling, the mechanical state of tissue-engineered constructs, and different external loading conditions, we are able to optimize and steer growth and remodeling via the rational design of scaffold geometry, thus creating long-term functioning grafts.

Bioreactor platforms provide a valuable means to study this interplay under tightly controlled conditions. We recently developed the Vertigro bioreactor, in which tissue-engineered cardiovascular constructs can be cultured under dynamic pressurization to mimic hemodynamic loading (Oomen et al., 2017; van Kelle et al., 2017). Importantly, the platform also allows for nondestructive mechanical and geometric characterization of tissue-engineered constructs during culture, allowing us to monitor the mechanical and geometric state of these constructs over time. In previous studies, this platform was limited to applying isotropic deformation, whereas most cardiovascular tissues, including heart valves and blood vessels, are deformed in an anisotropic manner (Zhou and Fung, 1997; Billiar and Sacks, 2000). We therefore implemented anisotropic deformation in the Vertigro bioreactor by introducing an elliptical scaffold geometry that was designed using finite element analysis. With the same approach, circular control scaffolds were designed to undergo isotropic deformation of similar magnitude. In this way, we aimed to characterize how initial scaffold geometry, resulting in either anisotropic or isotropic deformation, affects tissue growth and remodeling, and the evolution of the mechanical state and geometry of tissue-engineered constructs over time. Elliptical and circular tissue-engineered constructs were dynamically loaded at 1 Hz with a peak pressure of 6 kPa for 14 days in the Vertigro bioreactor. The mechanical state and geometry of the constructs were characterized on day 0, 4, 7, 11, and 14 of dynamic loading using ultrasound imaging and inverse finite element analysis. The mechanical state was characterized in terms of stress, strain, strain energy density, and stiffness in the central 50% of each construct. After 14 days of dynamic loading, the final tissue structure and composition were assessed. We found that the initial scaffold geometry-induced mechanical state of the tissue-engineered constructs dictated the evolution of their mechanical state and geometry over time, as well as their final structural organization. These results demonstrate that the initial mechanical state of tissue-engineered constructs plays an important role in determining the outcome of tissue growth and remodeling *in vitro*.

## 2 Methods

### 2.1 Model-Driven Design of Initial Scaffold Geometry to Induce Anisotropic Deformation

**FIGURE 1:**
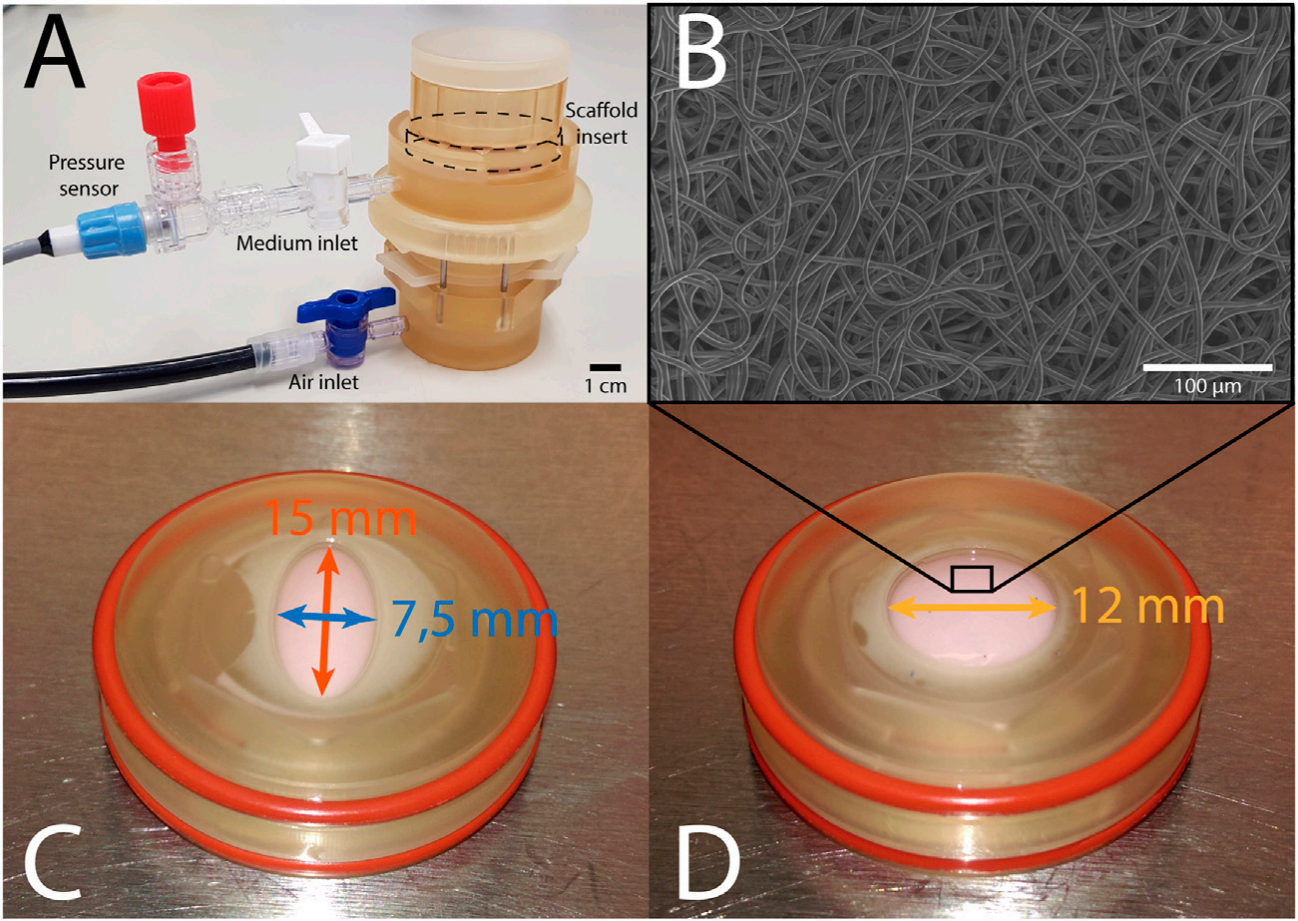
**(A)** The Vertigro bioreactor. **(B)** Scanning electron microscopy image of the scaffold prior to seeding. **(C)** Elliptical construct in insert measuring 7.5 mm (blue) by 15 mm (red). **(D)** Circular construct in insert with a 12 mm diameter (yellow).

The bioreactor (Figure 1A) features two chambers that are separated by a deformable membrane. Tissue-engineered constructs, mounted in inserts (Figures 1C, D), can be cultured in the top chamber, while being dynamically loaded via air pressurization of the bottom chamber. The pressure in the top chamber is monitored real-time, and constant maximum pressure is maintained via a custom computer-driven feedback system (van Kelle et al., 2017). This feedback system adjusts the air pressurization of the bottom chamber continuously to ensure the target pressure in the top chamber is achieved. In this bioreactor system, an axisymmetric insert geometry leads to isotropic in-plane deformation in the construct. In order to induce anisotropic deformation within these samples, elliptical inserts were developed, where the main axes measured 7.5 and 15 mm, respectively (Figure 1C). Circular constructs with a diameter of 12 mm were used as isotropic controls (Figure 1D). These dimensions were selected based on finite element modeling in Abaqus FEA (Dassault Systèmes Simulia, Providence, RI, United States) of pressurized circular and elliptical tissue-engineered constructs with varying dimensions. With the selected shapes and dimensions, anisotropic and isotropic deformation was achieved with similar maximum deformation and stress. Due to the symmetry of both shapes, only a quarter of each construct was modeled. Nodes on the outer edge of the constructs were fully encastered, while symmetry boundary conditions were applied on the inner edges. A pressure of 6 kPa was applied from underneath the tissue to simulate mechanical loading in the bioreactor. The constructs were modeled using a fiber-reinforced material model (Oomen et al., 2017), consisting of an isotropic matrix and a fibrous network. The Cauchy stress (***σ***) was determined via a strain energy density function Ψ as a function of a material parameter set *ξ*:

$$\sigma =\frac{2}{J}F\cdot \frac{\partial \Psi \left(\xi \right)}{\partial C}\cdot F^{\text{T}}$$ (1)

with **F** the deformation gradient tensor, **C** = **F** ^T^ ⋅**F** the right Cauchy-Green deformation tensor and the Jacobian *J* = det(**F**). Ψ was split in two components, the matrix part Ψ_*m*_ and the fibrous part Ψ_*f*_:

$$\Psi =\left(1-\Phi_{f}\right)\Psi_{m}+\Phi_{f}\Psi_{f}$$ (2)

in which the total fiber volume fraction Φ_*f*_ was set at 0.5 (Driessen et al., 2007). The isotropic matrix part was modeled as a Neo-Hookean material:

$$\Psi_{m}=\frac{\kappa }{2}\ln^{2}\left(J\right)+\frac{\mu }{2}\left(I_{1}-3-2\ln\left(J\right)\right)$$ (3)

with bulk modulus *κ*, shear modulus *μ* and the first invariant of the right Cauchy-Green deformation tensor *I*_1_ = **C**: **I**. The fibrous component was modeled with a discrete number of fibers in the plane of the construct. Each individual fiber (*i*) was associated with a vector $e_{f0}^{i}$ (equally-spaced in the reference configuration) that represents its direction within the tissue plane, and a volume fraction $\phi_{f}^{i}$ that is described by a Gaussian function:

$$\phi_{f}^{i}=A\exp\left(\frac{-{\left(\gamma^{i}-\alpha \right)}^{2}}{\beta^{2}}\right).$$ (4)

Here, the angle of the main fiber direction *α* is set to 0 (parallel to the long axis of the elliptical constructs), the angle *γ* ^*i*^ defines the orientation of a fiber *i* with respect to *α*, *β* represents the fiber dispersity, and *A* is a normalization factor to ensure that the sum of individual fiber fractions equals Φ_*f*_. The contribution of each fiber to the total strain energy was described by a non-linear model (Driessen et al., 2007; Oomen et al., 2018) to account for its strain-stiffening behavior, where the total strain energy density of the fibrous component Ψ_*f*_ is the sum of the strain energy densities of all individual fibers $\Psi_{f}^{i}$ , and:

$$\Psi_{f}^{i}=\frac{k_{1}}{2k_{2}}\left(\exp\left[k_{2}\left\langle {\left(\lambda^{i}\right)}^{2}-1\right\rangle \right]-k_{2}\langle {\left(\lambda^{i}\right)}^{2}-1\rangle -1\right)$$ (5)

with material parameters *k*_1_ and *k*_2_, the squared elastic fiber stretch ${\left(\lambda^{i}\right)}^{2}=C:\left(e_{f0}^{i}⊗e_{f0}^{i}\right)$ , and Macaulay brackets (⟨◦⟩) to enforce that the fibers only resist tension. The material parameters were set to resemble the material properties of an isotropic polycaprolactone-bisurea (PCL-BU) scaffold, which were determined using a BioTester (CellScale, Waterloo, Canada): *μ* = 1.45 ⋅10^2^ kPa, *k*_1_ = 1.32 ⋅10^7^ kPa, *k*_2_ = 2.64 ⋅10^–5^, and *β* = 100 to enforce an isotropic scaffold fiber distribution. The bulk modulus was estimated via $\kappa =\frac{2\mu \left(1+\nu \right)}{3\left(1-2\nu \right)}$ with shear modulus *μ* and an assumed Poisson’s ratio *ν* of 0.495.

### 2.2 Tissue Culture in the Vertigro Bioreactor

**FIGURE 2:**
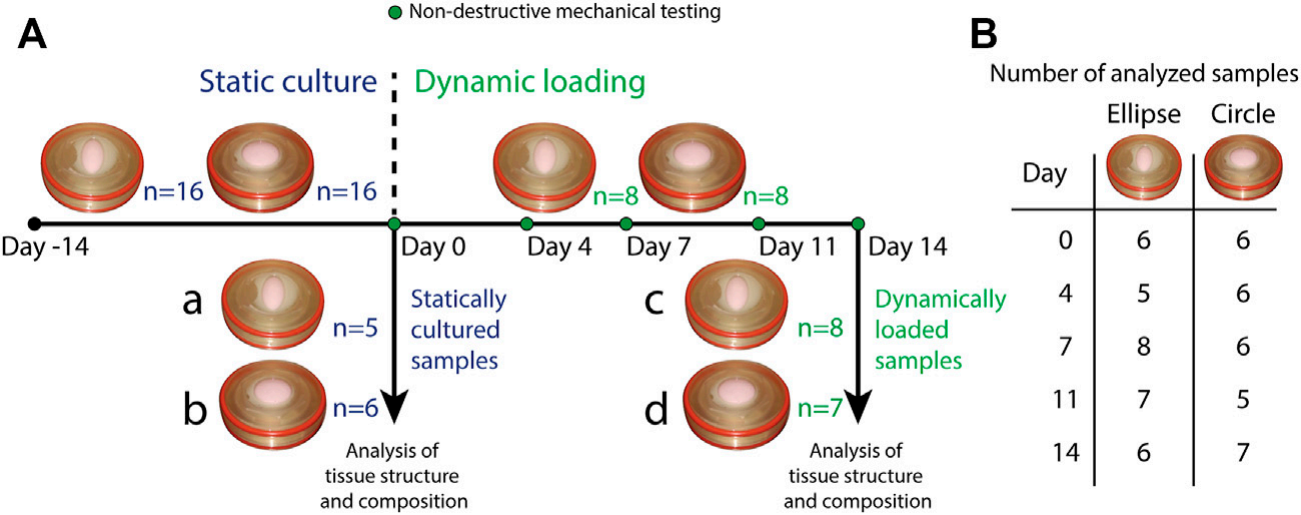
**(A)** Overview of the experimental design. All samples (*n* = 16 for both geometries) underwent static culture for 14 days. During static culture, three elliptical and two circular constructs detached from the insert and were therefore omitted from the analyses. After static culture, five elliptical and six circular statically cultured constructs were sacrificed for analysis of structure and composition (a. statically cultured elliptical constructs, b. statically cultured circular constructs). The remaining eight constructs per geometry were dynamically loaded for 14 days. During these 14 days, constructs were mechanically characterized on day 0, 4, 7, 11, and 14. During dynamic loading, one circular construct detached from the insert and was omitted as well. After dynamic loading, the remaining samples (8 elliptical and seven circular constructs) were also sacrificed for analysis of structure and composition (c. dynamically loaded elliptical constructs, d. dynamically loaded circular constructs). **(B)** Overview of the number of mechanically characterized samples per construct geometry during dynamic loading. Numbers vary per day because during some measurements, pressure data was found to be improperly calibrated. These samples were therefore omitted from the analysis of that particular time point.

In total, 16 circular and 16 elliptical tissue-engineered constructs were prepared. Bare, 0.3 mm thick electrospun PCL-BU scaffolds (Wisse et al., 2006) with isotropically distributed fibers (fiber diameter of approximately 4 *μm*) (Figure 1B) were cut from a sheet using a cork borer, mounted in the inserts and sterilized using UV light. Following sterilization, inserts were submerged in 0.25 mg/mL L-ascorbic 2-phosphate acid (Sigma-Aldrich, St. Louis, MO, United States) supplemented standard culture medium (TE medium) and placed in an incubator overnight. Human vena saphena cells (HVSCs), which were previously characterized as contractile, matrix-producing myofibroblasts (Mol et al., 2005), were isolated from human vena saphena samples according to the Dutch guidelines for use of secondary materials. These cells were cultured up to passage seven in advanced Dulbecco’s Modified Eagle Medium (Invitrogen, Carlsbad, CA, United States), supplemented with 10% Fetal Bovine Serum (Greiner Bio One, Frickenhausen, Germany), 1% Glutamax (Invitrogen) and 1% penicillin/streptomycin (Lonza, Basel, Switzerland). After trypsinization, the cells were seeded in the sterilized PCL-BU scaffolds at a density of 15 million cells/cm^3^ using fibrin as a cell carrier (Mol et al., 2005). To allow tissue formation inside the scaffolds before dynamic loading, all constructs were statically cultured for 14 days in TE medium in an incubator at 37 °C, 100% relative humidity and 5% CO_2_, where medium was changed twice a week. After 14 days of static culture, five elliptical and six circular constructs were sacrificed for analysis of structure and composition. These samples served as day 0 control samples for the other eight elliptical and circular constructs that were then dynamically loaded for 14 days in the bioreactor at a peak pressure of 6 kPa applied at a rate of 1 Hz. After dynamic loading, the remaining samples were sacrificed as well for analysis of structure and composition. For an overview of the experimental design including sample sizes, Figure 2.

### 2.3 Monitoring the Evolving Mechanical State and Geometry of Dynamically Loaded Constructs

#### 2.3.1 Nondestructive Mechanical and Geometric Characterization

Throughout the period of dynamic loading, constructs were mechanically characterized in a nondestructive manner on day 0, 4, 7, 11 and 14 (Figure 2), using a previously developed method (Oomen et al., 2017; van Kelle et al., 2017). Briefly, a 12 MHz linear ultrasound transducer on a MyLab 70 Ultrasound system (LA435 probe, Esaote, Maastricht, Netherlands) was placed on top of the Vertigro bioreactor to image the cross-section of the construct along the diameter of the circular constructs and both the short and long axes of the elliptical constructs (Figure 1C). During ultrasound imaging, constructs were gradually pressurized up to 6 kPa in 10 s. Afterwards, ultrasound images were processed using a custom MATLAB (MathWorks, Natick, MA, United States) script to obtain the constructs’ cross-sectional profile as a function of pressure. In case of elliptical constructs, the profiles along both axes were interpolated by taking the average height of the apex of each image to obtain one mean profile. Additionally, construct thickness was determined by collecting raw radiofrequency data from non-pressurized constructs. Using MATLABs *findpeaks* algorithm, the local maxima, or peaks, were identified in the raw data. The two highest peaks were defined as the tissue borders. Average construct thickness was estimated based on the distance between these two peaks in 20 locations per construct.

#### 2.3.2 Estimation of Material Properties and Mechanical State

Using the construct’s cross-sectional profile as a function of pressure (from 0 to 6 kPa), the material properties were estimated via a two-step inverse method as described in Oomen et al., 2017. In brief: as a first step, an initial estimate of the material properties was made based on shell theory [see (Oomen et al., 2017) for further details] and the construct’s cross-sectional profile as a function of pressure. In the second step, a full inverse finite element method was employed to obtain a more accurate estimate. Constructs were modeled using the same fiber-reinforced material model as described in Section 2.1.

In the second step, the final material parameters were estimated using MATLAB’s *lsqnonlin* function, starting from the initial estimates of the first step. This function incrementally adjusts the material parameter set [*ξ* = (*μ*, *k*_1_, *k*_2_, *β*)], compares the simulated cross-sectional profile of the construct with the construct cross-sectional profile obtained from the nondestructive mechanical testing, and repeats this process until the error *E* between these profiles, as a function of pressure (*p*), was minimized:

$$E\left(\xi \right)=\frac{1}{N_{p}N_{x}}\overset{N_{p}}{\underset{j=1}{\sum }}\overset{N_{x}}{\underset{k=1}{\sum }}\sqrt{z{\left(p_{j},x_{k},\xi \right)}^{\text{exp}}-z{\left(p_{j},x_{k},\xi \right)}^{\text{est}}}$$ (6)

where *N*_*p*_ is the number of pressure increments, and *N*_*x*_ the number of in-plane positions *x* along the construct profile. The inclusion of *β* in the material parameter set enables the model to account for mechanical anisotropy that may occur as a result of structural remodeling in the engineered constructs (Eq. (4)).

Following estimation of the material parameters in each timepoint, the Cauchy stress tensor, deformation gradient tensor, and the strain energy density at peak pressure were obtained from the nodes in the central 50% of the samples. Next, the principal stresses *σ*_*i*_ and their directions **n**_*i*_ were determined (with *i* = 1, 2, 3) by calculating the eigenvalues and eigenvectors, respectively, of the Cauchy stress tensor. The principal stretches *λ*_*i*_ were obtained from $\lambda_{i}=1/\sqrt{B^{-1}:\left(n_{i}⊗n_{i}\right)}$ , with *i* = 1, 2, 3 and **B** = **F** ⋅**F** ^T^ the left Cauchy-Green deformation tensor. The deformation gradient tensor **F** was provided by the finite element model. The maximum principal stress and stretch were analyzed for the circular constructs, while for the elliptical constructs the maximum and middle principal stress and stretch were found to approximately correspond with the in-plane stress and stretch along the short and long axis, respectively. The local tangent of the principal stress-stretch curves was determined at the peak pressure of 6 kPa. To quantify the overall mechanical state of a construct, these quantities were averaged for the inner 50% of each construct.

### 2.4 Analysis of Tissue Composition

After sacrificing a construct, one half was snap-frozen in liquid nitrogen, lyophilized overnight and pulverized using a Mikro-Dismembrator (Model S, Sartorius, Goettingen, Germany). The remains were digested for 16 h at 60°C using a papain digestion buffer. The amount of GAGs in the digest was quantified using an adapted protocol by Farndale et al. (1986), with a standard curve of chondroitin sulfate (Shark Cartilage, Sigma-Aldrich). Using a trans-4-hydroxyproline (Sigma-Aldrich) standard curve, the hydroxyproline (HYP) content (as a measure for collagen content) was quantified in accordance with the protocol of Huszar et al. (1980). Additionally, the DNA content was determined using a Qubit 2.0 fluorometer (Invitrogen). Lastly, a quarter of each construct was analyzed using the Fastin Elastin assay kit (Fastin, Biocolor) to quantify elastin content, which was normalized to construct wet weight.

### 2.5 Analysis of Tissue Structure

#### 2.5.1 Histology

From the remaining quarter of each construct, the two straight rims were cut off, thus obtaining an L-shaped part that coincides with the two semi-axes of the whole construct. This part was embedded in paraffin and sectioned through its thickness into slices of 7 μm. For the elliptical samples, sections were made along both the short and long axes. Part of these embedded sections were stained for general tissue structure [Hematoxylin and Eosin (HE)], and collagen [Picrosirius red (PR)], and subsequently imaged using a brightfield microscope (Axio Observer Z1, Carl Zeiss AG, Oberkochen, Germany). On the remaining sections, immunohistochemistry was performed to visualize (tropo)elastin, alpha-smooth muscle actin (*α*-SMA), as well as collagen type I and III. These sections were imaged using an Axiovert 200M microscope (Zeiss).

#### 2.5.2 In-Plane Collagen Orientation

The remaining part of each construct was analyzed for the in-plane collagen fiber orientation. Firstly, each sample was stained for collagen using a fluorescent CNA35 probe (1 h incubation time) (Boerboom et al., 2007). Secondly, tile scans (excitation 488 nm, emission 520 nm, magnification ×10, maximum 60 *μ*m deep) of both the top and bottom side of stained samples were made using a confocal laser scanning microscope (TCS SP5X; Leica Microsystems, Wetzlar, Germany). Collagen fiber distributions were quantified in MATLAB (Frangi et al., 1998). In brief, tissue structure was analyzed based on the Hessian matrix. Fiber-like structures were identified by means of the Frangi Vesselness. The principal direction of a fiber was subsequently determined by decomposition of the Hessian matrix. In this way, a histogram with the fraction of collagen fibers oriented in each in-plane direction was obtained, which was parameterized by means of a normalized unimodal Gaussian distribution using previously described algorithms (van Haaften et al., 2018). This algorithm provided an estimate for the fraction of aligned fibers in the whole construct.

### 2.6 Statistics

Numerical data are presented as mean ± standard error of the mean. The longitudinal geometric and mechanical data were analyzed in RStudio (RStudio Team, Boston, United States) using a mixed linear effects model, which accounts for correlations between the repeated measurements and remains reliable in case of samples missing completely at random (Hothorn and Everitt, 2009), as is the case here. Post hoc analysis to test for significant differences between groups at the same timepoint and between timepoints within a single group was done using the *Estimated Marginal Means* function. Biochemical assay data between groups was compared with the multiple comparison procedure developed by Herberich et al. (2010) in R 3.5.0 (The R foundation, Vienna, Austria). With this procedure, no assumptions are necessary regarding the distribution, sample sizes or variance homogeneity. Differences were considered significant if *p* ≤ 0.05.

## 3 Results

### 3.1 Model-Driven Design of Initial Scaffold Geometry

The selected elliptical geometry resulted in stretch anisotropy, with higher stretch along the short compared to the long axis (Supplementary Figure S1 left and Supplementary Figure S2). As expected, the in-plane distribution of stretch was isotropic in the circular construct when pressurized (Supplementary Figure S1 right). Importantly, these designs resulted in similar stress-stretch at maximum pressure along the short axis of the elliptical construct and the circular construct (Supplementary Figure S2).

### 3.2 Different Patterns of Enlargement Depending on Initial Scaffold Geometry

**FIGURE 3:**
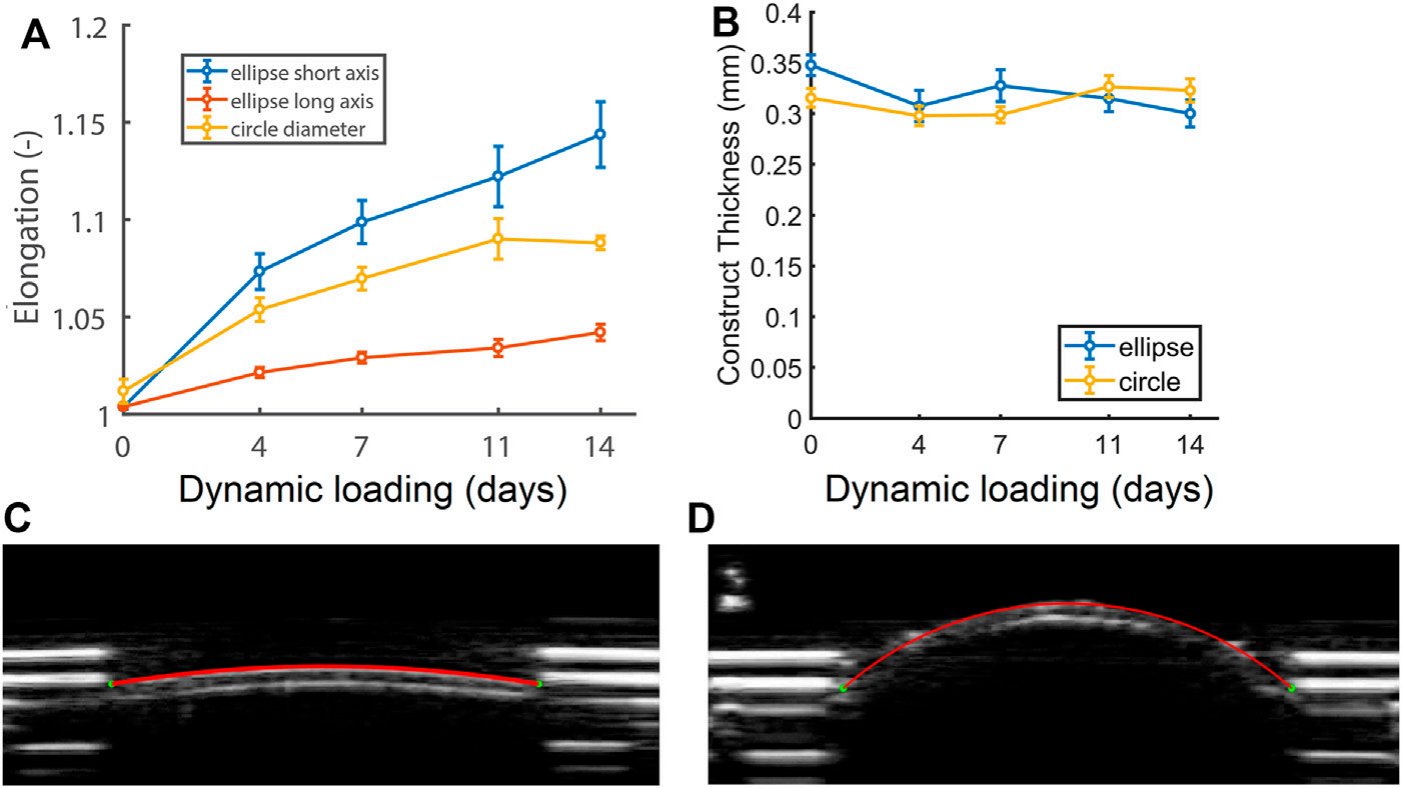
**(A)** Relative changes in cross-sectional length at zero pressure with respect to the initial length at the start of the experiment (*L*/*L*_0_) of the short (blue) and long (red) axis of the elliptical constructs and the diameter of the circular constructs (yellow), as well as **(B)** construct thickness during 14 days of dynamic loading. See supplemental material for *p*-values. **(C)** Ultrasound image of a circular construct at the start of dynamic loading, and **(D)** a dilated circular construct at the end of dynamic loading at zero pressure (*p* = 0).

At the start of the culture period, elliptical constructs measured 7.5 and 15 mm along the short and long axis respectively, and the circular constructs 12 mm in diameter (*L*_0_). Constructs enlarged during dynamic loading (Figure 3A), which was confirmed by the ultrasound data of a representative (circular) construct at zero pressure right before (Figure 3C) and on the last day of dynamic loading (Figure 3D). Elliptical constructs enlarged unevenly, with higher elongation along their short axis compared to their long axis (Figure 3A). The elongation along the elliptical constructs’ short axis and the cross-section of the circular constructs was similar until day 7, after which the elliptical constructs enlarged more than the circular constructs. Interestingly, the length of the cross-section of the circular constructs stabilized from day 11.

On day 0 of dynamic loading, elliptical constructs were slightly thicker than the circular constructs. From day 4, elliptical construct thickness decreased and remained similar to circular construct thickness for the rest of dynamic loading (Figure 3B).

### 3.3 Different Initial Scaffold Geometries Lead to Distinct Mechanical States

**FIGURE 4:**
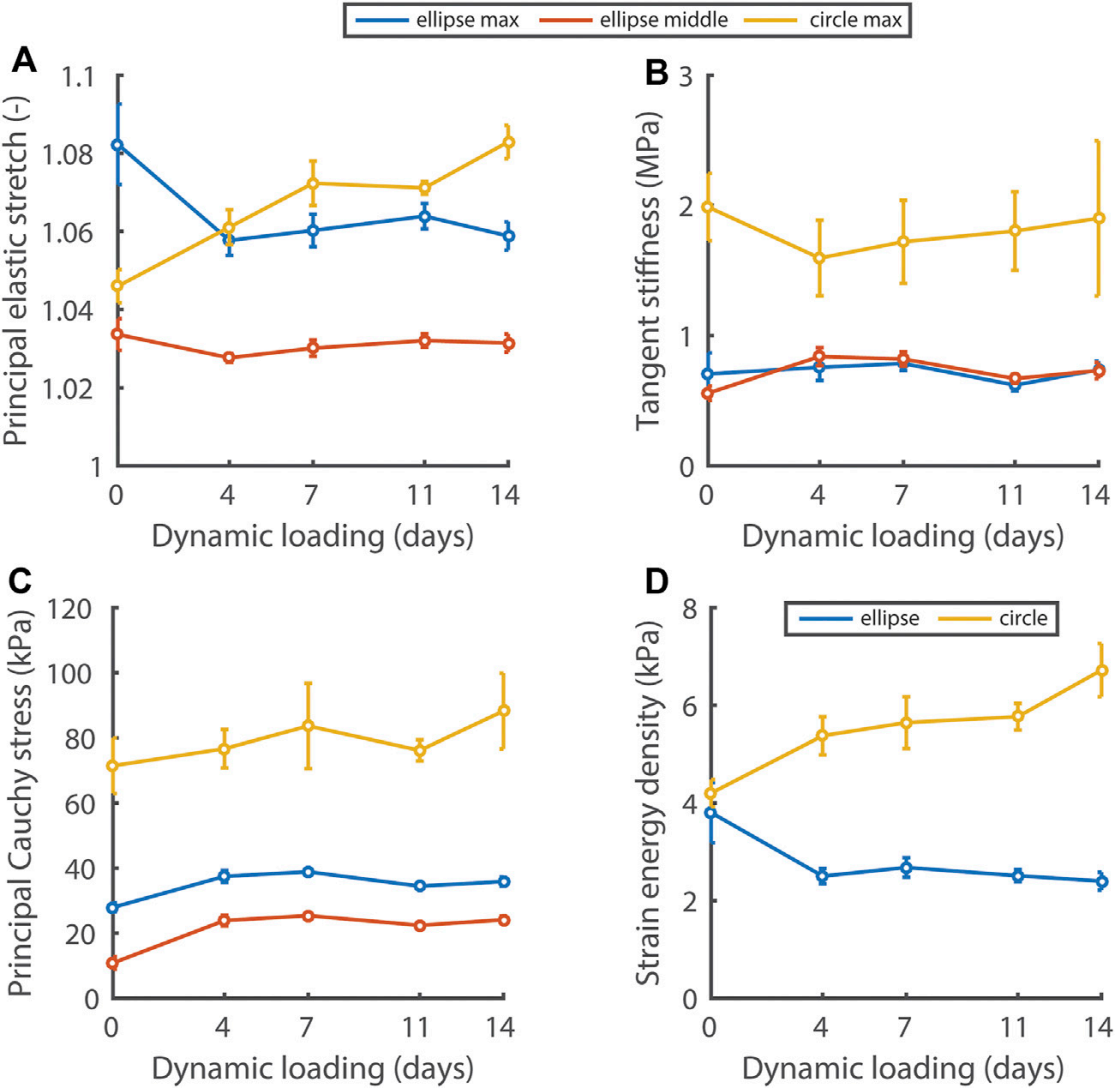
Evolution of the principal elastic stretch **(A)**, tangent stiffness of the principal stress-stretch curve **(B)**, principal Cauchy stress **(C)**, and strain energy density **(D)** at 6 kPa in the central 50% of elliptical and circular constructs during dynamic loading. In **(A–C)** the maximum and middle principal component for the elliptical constructs and the maximum component for the circular constructs are given. In the ellipses, the maximum and middle principal components of the Cauchy stress and stretch are directed along the short and long axis approximately. See supplemental material for *p*-values.

Using nondestructive mechanical testing and inverse finite element analysis, we monitored the evolution of four mechanical quantities in the elliptical and circular constructs (Figure 4). Interestingly, different mechanical states were observed between constructs, depending on the initial scaffold geometry. On day 0 of dynamic loading, all quantities except the strain energy density were already at different levels between the different construct geometries. After 4 days of dynamic loading, a stretch and strain energy density equilibrium was established and maintained in the elliptical constructs, whereas these quantities continuously increased in the circular constructs during dynamic loading (Figures 4A, D). The tangent stiffness (Figure 4B) and the maximum/middle principal Cauchy stress (Figure 4C), were at a higher level in the circular compared to the elliptical constructs, and did not change over time in neither construct type.

### 3.4 Initial Scaffold Geometry Influences Construct Elastin Content

**FIGURE 5:**
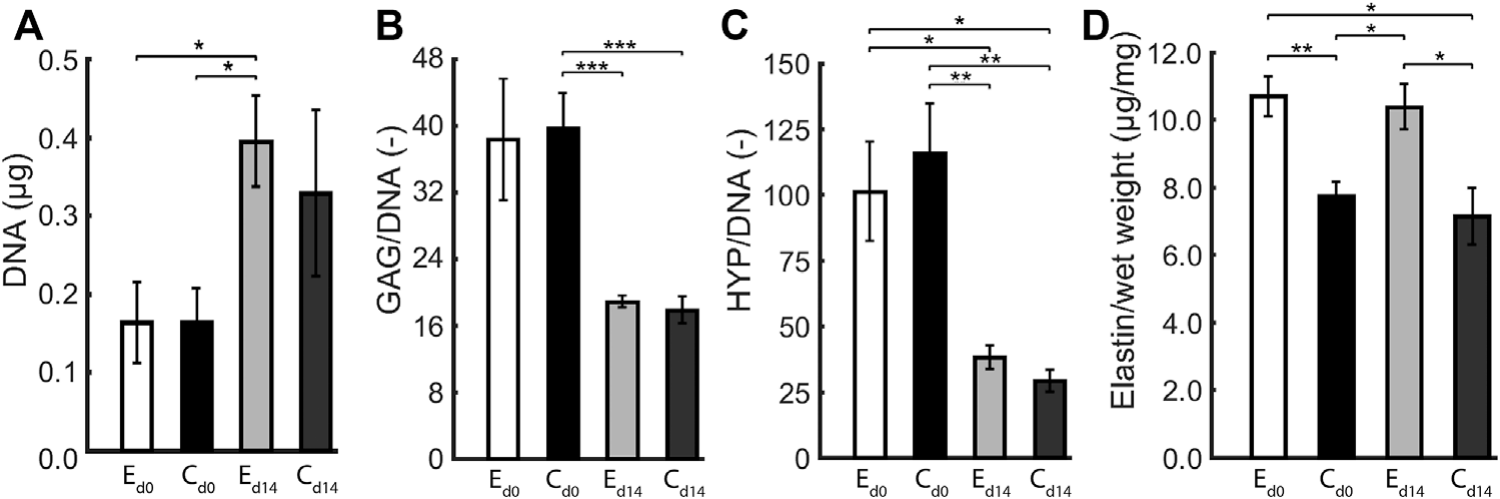
Average **(A)** DNA content, **(B)** GAG per DNA, **(C)** HYP per DNA, and **(D)** (tropo)elastin per wet weight in elliptical (E) and circular (C) constructs before (d0) and after 14 days (d14) of dynamic loading. One asterisk (*) represents *p* ≤ 0.05, two *p* ≤ 0.01, and three *p* ≤ 0.001.

The composition of statically and dynamically cultured constructs was quantified in terms of GAG, DNA, collagen (HYP), and (tropo)elastin content. There were no differences in the elliptical and circular constructs with respect to DNA content, GAG per DNA, and Hyp per DNA, both before and after dynamic loading (Figure 5). During dynamic loading, DNA content increased and GAG and Hyp per DNA decreased in all constructs in similar proportions, implying that collagen and GAG contents remained similar and cell numbers increased during dynamic loading. On the other hand, (tropo)elastin per construct wet weight was higher in the elliptical than the circular constructs, and remained similar during dynamic loading.

### 3.5 Anisotropic Deformation Results in Anisotropic In-Plane Collagen Organization

**FIGURE 6:**
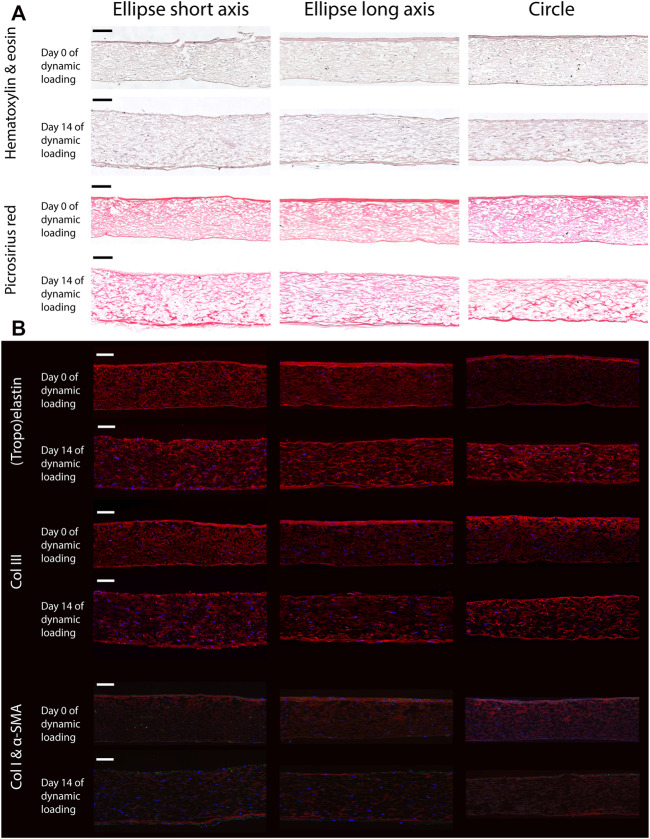
Representative histological **(A)** and immunohistochemical **(B)** cross-sections along the short and long axis of the elliptical constructs, and the diameter of the circular samples, before (day 0) and after (day 14) dynamic loading. Note that the bottom side of all samples coincides with the pressurized side in the bioreactor. Scale bars represent 100 *μ*m. **(A)** The top two rows show a Hematoxylin and Eosin staining to indicate the general tissue composition and cellular distribution. The bottom two rows feature a PR stain, depicting collagen fibers (red). **(B)** From top to bottom; elastin (red), collagen type 3 (red), collagen type 1 (red) and *α*-SMA (green). In all images cell nuclei are stained blue. Negative controls for immunohistochemical images can be found in Supplementary Figure S3.

Histology revealed that the elliptical and circular constructs were structurally similar. Additionally, the structure Along the short and long axis of the elliptical constructs were similar as well. The HE staining showed that the HVSCs were homogeneously distributed throughout the thickness of all constructs (Figure 6A). In both the HE and PR images, a dense tissue layer was visible on the top side of all statically cultured samples. Interestingly, after dynamic loading this layer was found on the opposite, pressurized side (Figure 6A).

**FIGURE 7:**
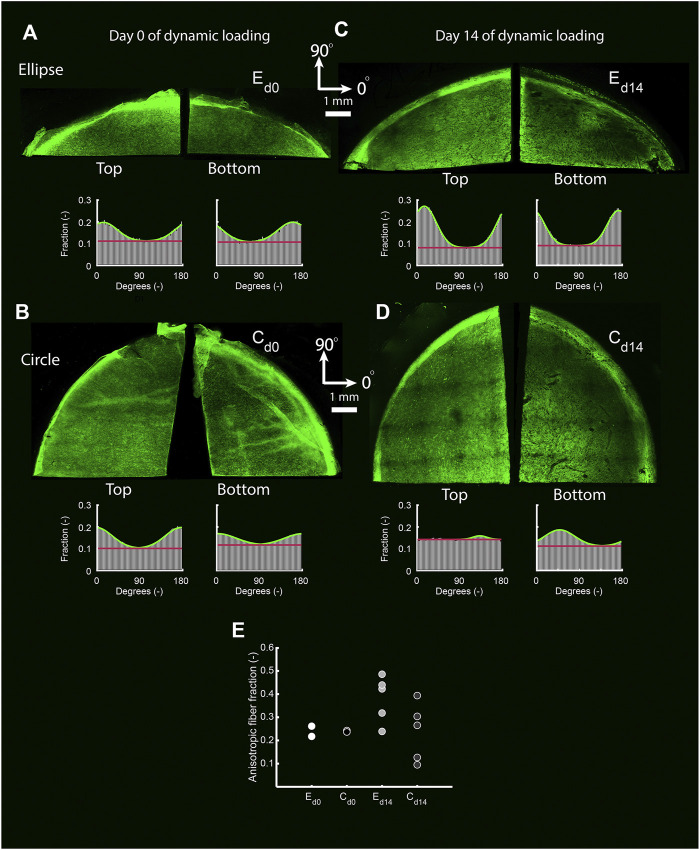
The top and bottom side of representative collagen-stained quarters of elliptical **(A,C)** and circular **(B,D)** constructs before (day 0) **(A,B)** and after (day 14) **(C,D)** dynamic loading. The histograms indicate the fraction of collagen fibers oriented in each direction for each image. Within histograms, the green line represents a Gaussian fit for the fiber distribution. The fiber fraction above the red baseline represents the anisotropic fiber fraction based on the Gaussian fit. **(E)** Average anisotropic fiber fraction of the top and bottom side of the pre-dynamic loading (d0) elliptical (E) and circular (C) constructs (both *n* = 2), and the post-dynamic loading (d14) elliptical and circular constructs (both *n* = 5).

In the statically cultured constructs, both tropoelastin aggregates and collagen type III were primarily found at the top and bottom surfaces, whereas after dynamic loading these were seen across the whole thickness (Figure 6B). A dense collagen type I layer was observed on top of the constructs after static culture, which after dynamic loading was present on the bottom instead. Only a few *α*-SMA-positive cells were found in the constructs, regardless of construct geometry and culture time (Figure 6B). On the other hand, the initial construct geometry in combination with dynamic loading had a clear effect on the in-plane structural collagen organization (Figure 7). Before dynamic loading (day 0), the fraction of anisotropic collagen fibers was similar in the circular and elliptical constructs (day 0) (Figures 7A,B,E). After dynamic loading (day 14), the average anisotropic fiber fraction was increased for the elliptical constructs whereas it remained similar for the circular constructs (Figures 7C–E). The presence of a small anisotropic collagen fiber fraction in the day 0 constructs and the circular day 14 constructs was also seen in the scaffold fibers (Supplementary Figure S4). The preferred orientations of the collagen and scaffold fibers in these samples coincided, suggesting that the scaffold fibers provided contact guidance cues to the cells that then produced collagen along their long axis, resulting in a collagen organization matching with the scaffold fiber directions.

## 4 Discussion

*In situ* tissue engineering relies on growth and remodeling to build a functional tissue from a non-living scaffold. Therefore, understanding, predicting, and directing growth and remodeling in tissue-engineered constructs is key to enable successful *in situ* tissue engineering strategies. To obtain control over growth and remodeling, we must explore how scaffold parameters, such as geometry, influence the interplay between tissue growth, remodeling, and the construct’s mechanical state, thereby ultimately enabling the rational design of scaffolds that promote functional growth and remodeling. To this end, here we demonstrated that initial scaffold geometry dictates the mechanical state of tissue-engineered constructs during short-term dynamic loading *in vitro*. Besides distinct mechanical states, we also observed differences in the final collagen organization of constructs depending on the initial scaffold geometry. As the biomechanical behavior of cardiovascular tissues effectively defines their functionality, these findings indicate that initial scaffold geometry can determine the short-term functionality of cardiovascular tissue-engineered constructs while the scaffold is still present in the construct. Additionally, all constructs dilated during dynamic loading regardless of initial scaffold geometry, a property that must be considered in the *in vivo* application of PCL-BU scaffolds, as ultimately the functionality of many cardiovascular tissues such as heart valves and blood vessels relies on their dimensions.

Our findings are in agreement with previous studies that reported distinct mechanical and geometric states in tissue-engineered constructs depending on initial scaffold thickness, both *in vitro* (van Kelle et al., 2018) and *in vivo* (Wu et al., 2021). Even microscale geometric properties, such as fiber alignment affect the short-term mechanical evolution of engineered constructs (Uiterwijk et al., 2020). Collectively, these results show that construct functionality is highly dependent on scaffold design.

We observed different mechanical equilibria in our constructs depending on the initial scaffold geometry. Additionally, the circular constructs appeared to reach geometric stability (i.e. equilibria in terms of thickness and in-plane dimensions), in contrast to the elliptical constructs. However, it remains uncertain to what extent these equilibria are a manifestation of a homeostasis that is established by the cells in these constructs, or whether the cells have little to no influence on the behavior of the construct due to the presence of the PCL-BU scaffold. We hypothesize that the latter is the case as there are no structural or compositional differences between the elliptical and circular constructs that clearly explain the different mechanical states. Possibly, the differences in evolution of the mechanical state can be explained by the initial scaffold geometry and permanent deformation that is known to occur in these scaffolds (van Kelle et al., 2018). Furthermore, it remains an open question if, once the scaffold starts to degrade, the observed mechanical and geometric equilibria would be maintained over time. Alternatively, the lack of mechanical stability (i.e. equilibria in terms of (all) mechanical quantities) or geometric stability in the circular and elliptical constructs, respectively, could be interpreted as a hallmark of adverse growth and remodeling. However, Drews and colleagues predicted computationally and showed experimentally that early stenosis can be spontaneously resolved in tissue-engineered vascular grafts *in vivo* (Drews et al., 2020). From this perspective, it could well be possible that on longer timescales the initial enlargement of our elliptical and circular constructs is reversed, and stretch and strain energy density equilibria are established in our circular constructs.

For future research, it would be interesting to monitor the mechanical state of constructs with degradable scaffolds, considering the profound effects that scaffold degradation can have on both collagen organization and the mechanical state of tissue-engineered constructs (de Jonge et al., 2014; Uiterwijk et al., 2020). Additionally, in our pressure-controlled bioreactor, the PCL-BU scaffolds could undergo unrestricted permanent deformation, which continuously altered the reference configuration of the constructs, and therefore could have influenced mechanically-driven tissue growth and remodeling through the impact that the reference configuration has on the obtained stresses and strains. Finally, the role of the immune system in matrix production and scaffold degradation *in situ* tissue-engineeing could be addressed in future work, for instance by including macrophages (Wissing et al., 2017) in tissue culture. Ultimately, the success of *in situ* tissue engineering strategies will depend on the interplay of all these factors.

There are several limitations to this study. For the elliptical constructs, two ultrasound measurements along the short and long axis were required at each time point. The data from both axes were interpolated, which added an uncertainty to the geometric and mechanical data of these constructs. Theoretically, the apex displacement from both measurements should be identical, although in practice this was not always exactly the case. This can be attributed to the fact that the ultrasound probe was manually positioned during the ultrasound measurements, and therefore was not perfectly aligned with the short and long axis. An effective solution to this problem would be the use of 3D echography, which allows the entire construct to be imaged at once, omitting the necessity of interpolation. Additionally, collagen orientation could only be quantified up to 60 *μ*m deep using confocal microscopy. As a solution, high frequency ultrasound imaging could be used to evaluate the collagen organization throughout the construct thickness, as performed by Riggin et al. (2014) in their study on rat tendons *in vivo*. Applying this technique in the current study would help to gain valuable insights into the structural collagen fiber adaptation process. Despite accounting for the different volumes of the elliptical and circular scaffolds at the start of culture by seeding constructs with the same cell density, the measured DNA content at end of culture was similar for the elliptical and circular constructs (Figure 5A) albeit the circular constructs had an ≈ 1.3 times larger volume. This implies that at the end of culture the cell density was ≈1.3 times higher in the elliptical constructs, possibly due to a difference in cell proliferation depending on the geometry of the scaffold. This could also explain the factor ≈1.3 higher density of elastin in the elliptical compared to the circular constructs (Figure 5D), assuming all cells produced a similar amount of elastin. Lastly, our engineered constructs displayed viscoelastic behavior and permanent deformation, while we only incorporated elastic behavior in our material model. To account for this limitation at least partially, we determined construct profiles non-invasively in reference state (at *p* = 0 kPa) and loaded state (at *p* = 6 kPa) for each individual construct on every day of experimental characterization, and incorporated these changes in reference configuration in our computational analyses. Additionally, we mechanically tested constructs with a single load cycle from 0 to 6 kPa in 10 s to minimize the influence of viscoelastic behavior.

## 5 Conclusion

In summary, we studied how anisotropic versus isotropic mechanical loading—as imposed by initial scaffold geometry—affects tissue growth and remodeling, and the evolution of the mechanical and geometric state in cardiovascular tissue-engineered constructs over time. Using a bioreactor that allows culturing of tissue-engineered constructs in combination with nondestructive mechanical testing, we found that initial scaffold geometry dictates how the mechanical state, geometry, and structural organization of tissue-engineered cardiovascular constructs evolve in the early phase of regeneration. These results demonstrate that mediating the initial mechanical state of tissue-engineered constructs via scaffold geometry might be an interesting approach to influence tissue growth and remodeling and thus modulate tissue outcomes.

## Data Availability

The raw data supporting the conclusion of this article will be made available by the authors, without undue reservation.
