# Supplementary material for: Scaffold Geometry-Imposed Anisotropic Mechanical Loading Guides the Evolution of the Mechanical State of Engineered Cardiovascular Tissues in vitro
Source: Front Bioeng Biotechnol. 2022 Feb 16;10:796452. doi: 10.3389/fbioe.2022.796452 (PMC8888825; doi:10.3389/fbioe.2022.796452)
Supplement: Supplementary file 1 [file DataSheet1.PDF]

SUPPLEMENTARY MATERIAL

Figure S1: Stretch at 6 kPa along the short and long axis of the elliptical (left) and circular bare scaffold (right).

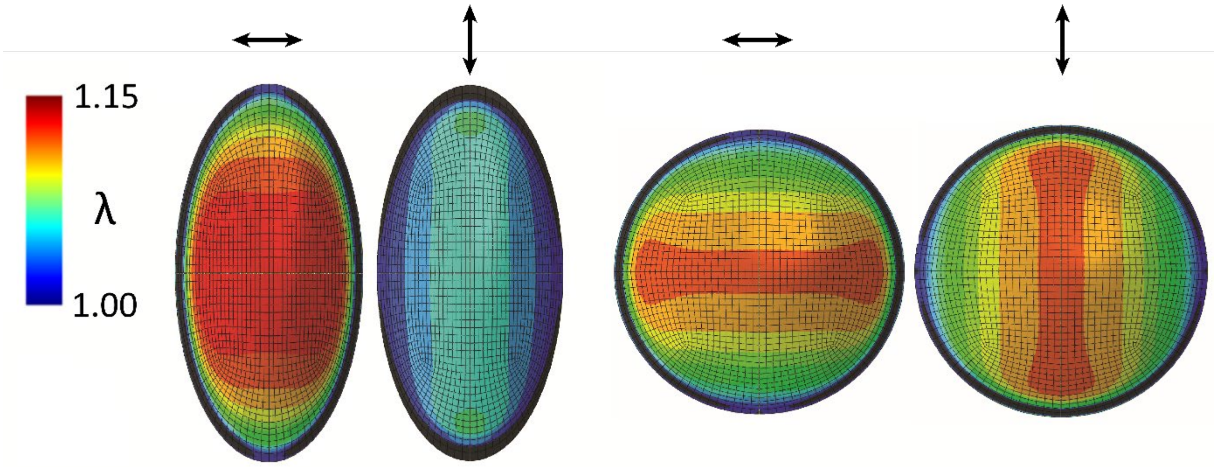

Figure S2: Cauchy stress versus stretch at the center of an elliptical and circular bare scaffold along the two main axes. In case of the circle this is the same line. All scaffolds had a thickness of 0.3 mm and were pressurized to 6 kPa.

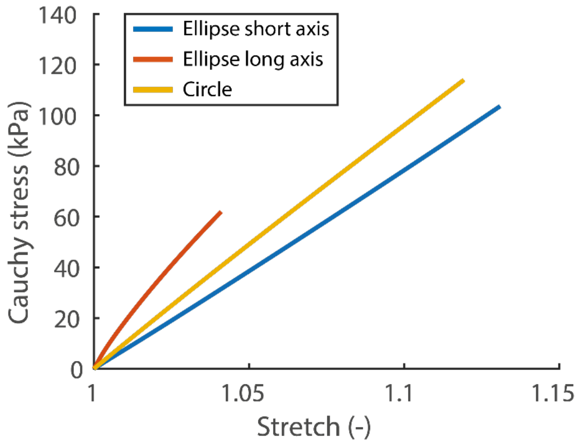

Figure S3: Negative controls of immunohistochemistry for (tropo)elastin (red), collagen type III (red), and collagen type I (red) and α-SMA (green), respectively. Scale bars represent 100 μm. In all images cell nuclei are stained blue.

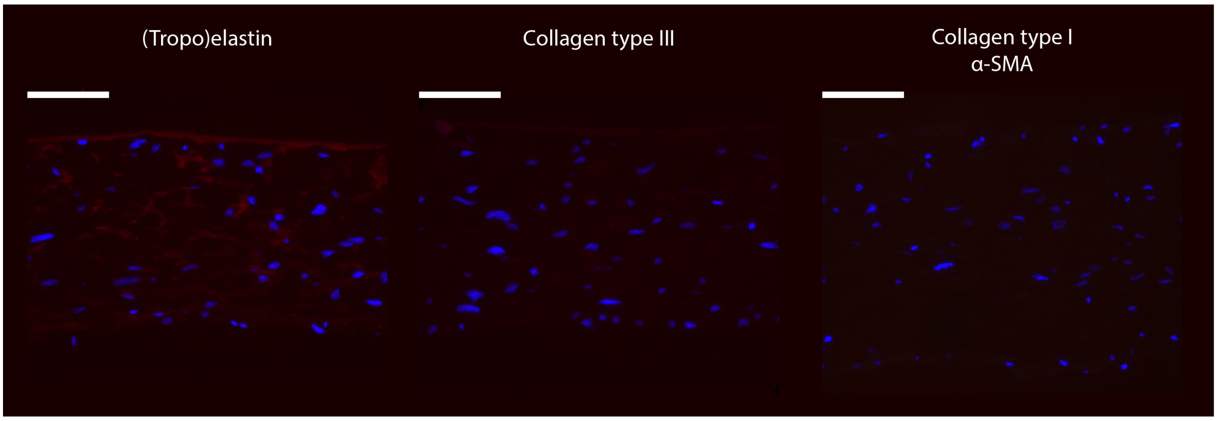

Figure S4: The top and bottom side of representative collagen-stained quarters of elliptical **(A and C)** and circular **(B and D)** constructs before (day 0) **(A and B)** and after (day 14) **(C and D)** dynamic loading. Scaffolds are visualized in the same samples by exploiting the autofluorescence properties of PCL-BU (excitation = 405 nm, emission = 460 nm). The histograms indicate the fraction of collagen or scaffold fibers oriented in each direction for each image. Within histograms, the green line (collagen) or blue line (scaffold) represents a Gaussian fit for the fiber distribution. The fiber fraction above the red baseline represents the anisotropic fiber fraction based on the Gaussian fit. Average anisotropic collagen **(E)** and scaffold **(F)** fiber fraction of the top and bottom side of the pre-dynamic loading (d0) elliptical (E) and circular (C) constructs (both n=2), and the post-dynamic loading (d14) elliptical and circular constructs (both n=5).

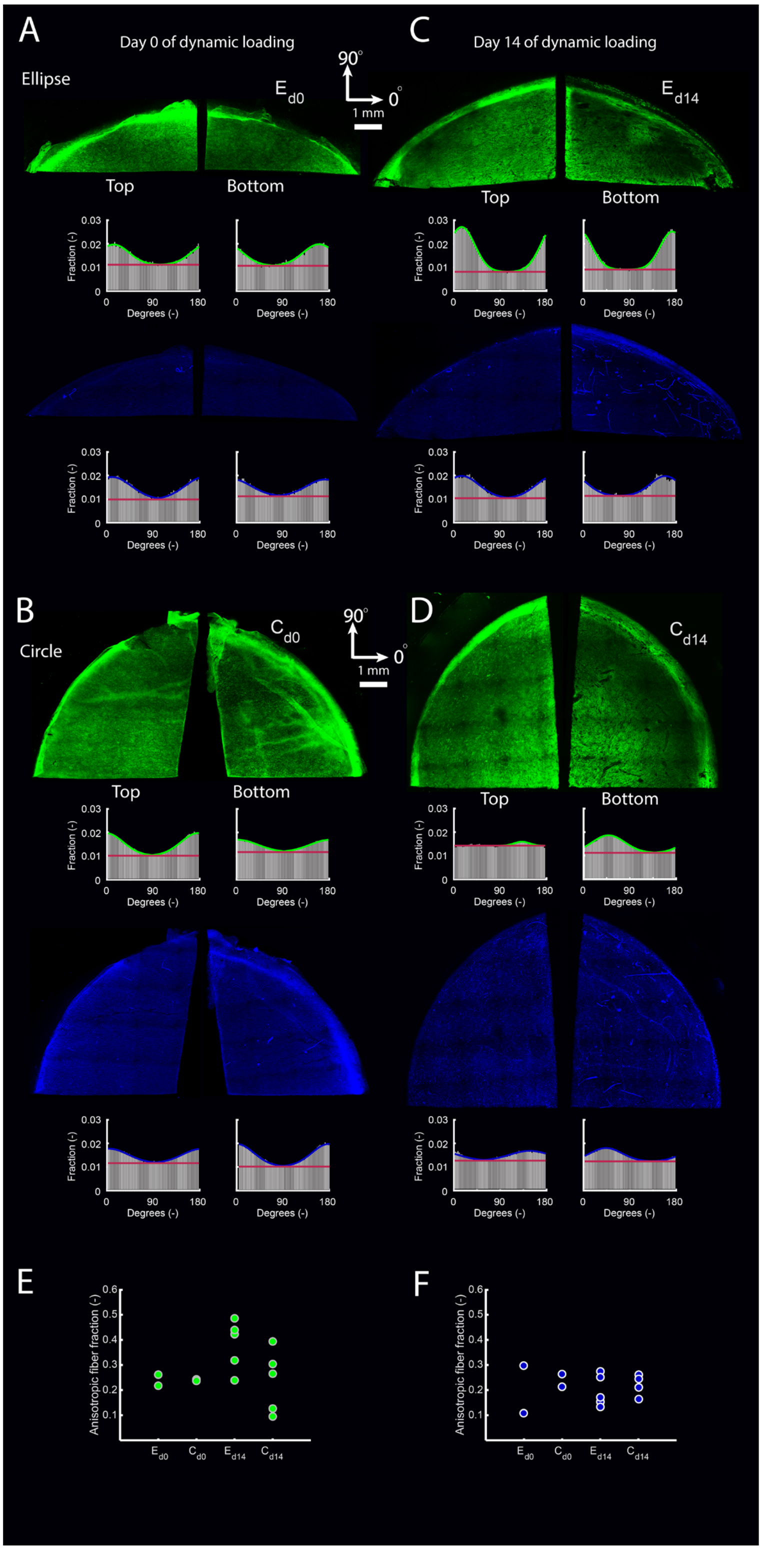

Linear mixed effects model p-values

Elongation

Differences between timepoints within a group

Ellipse long axis	Day 4	Day 7	Day 11	Day 14
Day 0	0.2734	0.0194	0.0026	0.0001
Day 4		0.8620	0.5159	0.0710
Day 7			0.9735	0.4475
Day 11				0.8230

Ellipse short axis	Day 4	Day 7	Day 11	Day 14
Day 0	0.0001	0.0001	0.0001	0.0001
Day 4		0.0193	0.0001	0.0001
Day 7			0.0715	0.0001
Day 11				0.0715

Circle	Day 4	Day 7	Day 11	Day 14
Day 0	0.0001	0.0001	0.0001	0.0001
Day 4		0.5835	0.0009	0.1032
Day 7			0.1032	0.1502
Day 11				0.8230

Differences between groups per timepoint

	Ellipse short axis / ellipse long axis	Ellipse short axis / circle	Ellipse long axis / circle
Day 0	0.9817	0.7126	0.8093
Day 4	0.0001	0.2151	0.0074
Day 7	0.0001	0.0175	0.0016
Day 11	0.0001	0.0183	0.0001
Day 14	0.0001	0.0001	0.0002

Thickness

Differences between timepoints within a group

Ellipse	Day 4	Day 7	Day 11	Day 14
Day 0	0.0084	0.4255	0.0550	0.0005
Day 4		0.5031	0.9733	0.9485
Day 7			0.8648	0.1368
Day 11				0.6621

Circle	Day 4	Day 7	Day 11	Day 14
Day 0	0.5701	0.5701	0.7826	0.9594
Day 4		1	0.0645	0.1891
Day 7			0.6545	0.1891
Day 11				0.9910

Differences between groups per timepoint

	Ellipse / circle
Day 0	0.0424
Day 4	0.5351
Day 7	0.0730
Day 11	0.4567
Day 14	0.1554

Elastic stretch

Differences between timepoints within a group

Ellipse long axis	Day 4	Day 7	Day 11	Day 14
Day 0	0.64	0.9630	0.9576	0.9985
Day 4		0.9047	0.9325	0.7724
Day 7			1	0.9954
Day 11				0.9929

Ellipse short axis	Day 4	Day 7	Day 11	Day 14
Day 0	0.0001	0.0001	0.0001	0.0001
Day 4		0.8796	0.4341	1
Day 7			0.8811	0.8073
Day 11				0.2932

Circle	Day 4	Day 7	Day 11	Day 14
Day 0	0.0005	0.0001	0.0001	0.0001
Day 4		0.0979	0.1213	0.0001
Day 7			1	0.1114
Day 11				0.1539

Differences between groups per timepoint

	Ellipse short axis / ellipse long axis	Ellipse short axis / circle	Ellipse long axis / circle
Day 0	0.0001	0.0001	0.0624
Day 4	0.0001	0.5621	0.0001
Day 7	0.0001	0.0389	0.0001
Day 11	0.0001	0.2398	0.0001
Day 14	0.0001	0.0001	0.0001

Strain energy density

Differences between timepoints within a group

Ellipse	Day 4	Day 7	Day 11	Day 14
Day 0	0.0130	0.0251	0.0068	0.0024
Day 4		0.9746	1	0.9982
Day 7			0.9747	0.8619
Day 11				0.9944

Circle	Day 4	Day 7	Day 11	Day 14
Day 0	0.0117	0.0022	0.0015	0.0001
Day 4		0.9835	0.9479	0.0089
Day 7			0.9995	0.0486
Day 11				0.1315

Differences between groups per timepoint

	Ellipse / circle
Day 0	0.2151
Day 4	0.0001
Day 7	0.0001
Day 11	0.0001
Day 14	0.0001

Cauchy stress

Differences between timepoints within a group

Ellipse long axis	Day 4	Day 7	Day 11	Day 14
Day 0	0.3849	0.1834	0.4293	0.3199
Day 4		0.9997	0.9995	1
Day 7			0.99	0.9998
Day 11				0.999

Ellipse short axis	Day 4	Day 7	Day 11	Day 14
Day 0	0.6739	0.4555	0.8595	0.7796
Day 4		0.9998	0.9918	0.9992
Day 7			0.9537	0.9902
Day 11				0.9996

Circle	Day 4	Day 7	Day 11	Day 14
Day 0	0.9362	0.2804	0.9096	0.0600
Day 4		0.7527	0.999	0.3425
Day 7			0.8496	0.9743
Day 11				0.4808

Differences between groups per timepoint

	Ellipse short axis / ellipse long axis	Ellipse short axis / circle	Ellipse long axis / circle
Day 0	0.0796	0.0001	0.0001
Day 4	0.1969	0.0001	0.0001
Day 7	0.0826	0.0001	0.0001
Day 11	0.1737	0.0001	0.0001
Day 14	0.2402	0.0001	0.0001

Tangent stiffness

Differences between timepoints within a group

Ellipse long axis	Day 4	Day 7	Day 11	Day 14
Day 0	0.8902	0.8721	0.9919	0.9686
Day 4		1	0.9821	0.9976
Day 7			0.9809	0.9983
Day 11				0.9994

Ellipse short axis	Day 4	Day 7	Day 11	Day 14
Day 0	0.9998	0.9948	0.9990	0.9999
Day 4		1	0.9919	1
Day 7			0.9710	0.9999
Day 11				0.9916

Circle	Day 4	Day 7	Day 11	Day 14
Day 0	0.6172	0.9572	0.9992	0.9999
Day 4		0.9561	0.8093	0.6849
Day 7			0.9939	0.9783
Day 11				1

Differences between groups per timepoint

	Ellipse short axis / ellipse long axis	Ellipse short axis / circle	Ellipse long axis / circle
Day 0	0.8920	0.0003	0.0001
Day 4	0.9681	0.0350	0.0663
Day 7	0.9911	0.0023	0.0035
Day 11	0.9825	0.0002	0.0004
Day 14	0.9996	0.0003	0.0003
